# Contribution of immune cells and cytokines to radiation-induced injury: the key roles and potential targets

**DOI:** 10.3389/fimmu.2026.1785418

**Published:** 2026-04-28

**Authors:** Yanan Wu, Yuanjing Tian, YingYing Zhang, Pingping Hu, Lili Qiao, Guodong Deng, Ning Liang, Yan Zhang, Jiandong Zhang

**Affiliations:** 1 Department of Oncology, The First Affiliated Hospital of Shandong First Medical University & Shandong Provincial Qianfoshan Hospital, Shandong Lung Cancer Institute, Jinan, Shandong, China; 2 Department of Oncology, Heze Hospital Affiliated to Shandong First Medical University, Heze, Shandong, China; 3 Department of Oncology, Shandong First Medical University & Shandong Academy of Medical Sciences, Jinan, Shandong, China; 4 Department of Oncology, Shandong University of Traditional Chinese Medicine, Jinan, Shandong, China; 5 Department of Diagnostics, Medical Integration and Practice Center, Cheeloo College of Medicine, Shandong University, Jinan, Shandong, China; 6 Shenzhen Research Institute, Shandong University, Shenzhen, Guangdong, China

**Keywords:** cytokines, immune cells, immunomodulation, injury, radiation

## Abstract

Despite the useful applications of ionizing radiation in daily life, the heightened risks of radiation-induced injury have emerged as a significant challenge. Immune cells and the associated cytokines play crucial roles in orchestrating the inflammatory and repair processes that follow radiation exposure. These immune responses contribute to both the beneficial and detrimental outcomes of radiotherapy. Cytokines, in particular, regulate the recruitment and activation of immune cells, thereby influencing the extent and nature of tissue damage. By understanding the mechanisms underlying the interactions between immune cells and cytokines in radiation-induced injury, we can identify potential targets for therapeutic interventions. This article provides insights into the mechanisms underlying radiation-induced injury and highlights opportunities for enhancing the efficacy and safety of radiotherapy through immune-based interventions.

## 1 Introduction

In recent years, the heightened risks of IR (ionizing radiation) exposure have become a significant threat due to the tremendous rise in radiation applications in daily life. Ionizing radiation, a type of energy, is released by particles (α, β or neutrons) or electromagnetic waves (X-rays or γ-rays). Exposure to IR from both natural and artificial sources can result in potentially fatal outcomes. Natural radiation originates from various sources, including lightning and radioactive elements present in the air, water, and soil. One prominent source of artificially generated ionizing radiation is from the utilization of radiation in medical procedures, including medical imaging with X-rays, nuclear medicine, and radiotherapy. While exposure is targeted at a specific site, neighboring healthy tissues may also suffer consequences. In addition, other sources encompass spillage of nuclear material and potential terrorist activity involving radiologic weapons which can cause widespread fear and pose heightened risks to society (1–4). In this review, we specifically focus on radiation injury arising from radiotherapy, causing profound suffering and diminishes quality of life for countless patients.

IR can result in immediate damage to various cellular organelles and components, specifically targeting DNA, RNA, mitochondria, and the cellular membrane, with the rapid protein modification and the production of free radicals and reactive oxygen. The resulting repair of the damage or cell death then triggers a series of inflammatory response (5). IR interacts with the immune system in numerous ways, altering both the numbers and functions of immune cells. These damaged immune cells subsequently release large quantities of cytokines, which further intensify the ongoing inflammatory response. Both the damaged immune cells and expression of cytokines play crucial roles in the development of radiation-induced injury (6).

Exposure to high doses of radiation can cause severe and irreparable harm to various organ systems, especially those composed of rapidly dividing cells, such as the hematopoietic system, skin, and gastrointestinal tract. However, there are only a limited number of agents that have received approval from the U.S. Food and Drug Administration (FDA) specifically for use in treating individuals exposed to radiation (7). Exploring the role of the immune system in radiation-induced injury can facilitate the restoration of the immune system, enhance tissue repair, and ultimately improve survival rates. Accordingly, this article aims to provide a comprehensive review of factors and associated mechanisms contributing to the development of radiation injury, including the interactions of different parts of the immune system and the potential therapeutic opportunities. Elucidating the mechanisms underlying radiation-induced immune system injury and repair could pave the way for the development of novel medical countermeasures aimed at restoring immune balance.

## 2 Radiation-induced organ injury

**Figure 1:**
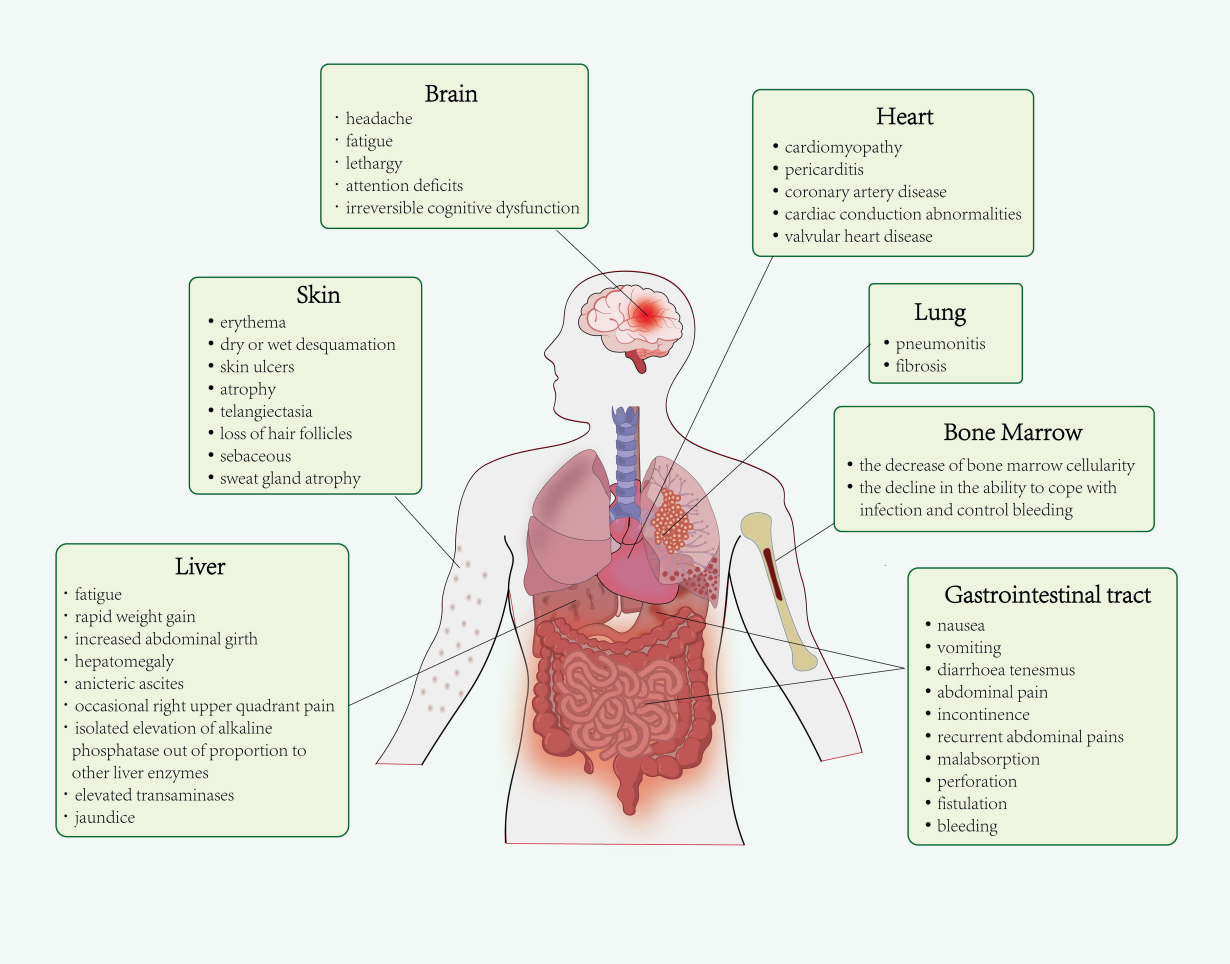
Radiation-induced organ injury. Ionizing radiation can induce radiation-induced injury affecting various tissue types involving bone marrow (BM), skin, heart, lung, brain, liver, gastrointestinal tract and so on.

Based on the absorbed radiation doses and the specific areas exposed, ionizing radiation can induce radiation-induced injury affecting various tissue types, including bone marrow (BM), skin, heart, lung, brain, liver, gastrointestinal tract, among others (**Figure 1**).

The most sensitive tissues, particularly those with rapidly dividing cells such as bone marrow, are more vulnerable to harm (8). Bone marrow exposure to radiation drastically decreases the production of leukocytes, particularly neutrophils and platelets, thereby compromising the ability to efficiently respond to infections and control bleeding (9). In addition to bone marrow, the skin is one of the tissues most susceptible to ionizing radiation due to the presence of continuously proliferating and differentiating cells. The pathophysiological changes in radiation-induced skin injury (RSI) are characterized by early erythema and dry or wet desquamation that occur shortly, as well as chronic skin ulcers, atrophy, telangiectasia, loss of hair follicles, sebaceous and sweat gland atrophy. The RSI may result from the depletion of stem and progenitor cells in the basal and dermal layers, along with complex multi-cellular interactions mediated by various inflammatory factors, ultimately resulting in fibrotic processes. Further, previous studies showed that radiation-induced heart disease (RIHD) appears several months to years following thoracic radiotherapy (10). The pathological processes of RIHD involve endothelial dysfunction, inflammation, fibrosis, and hypertrophy. RIHD manifests a range of cardiac dysfunction, including cardiomyopathy, pericarditis, coronary artery disease, cardiac conduction abnormalities, and valvular heart disease (11, 12). The lung is categorized as one of several moderately radiation-sensitive organs. Clinical manifestations of radiation-induced lung injury (RILI) encompass two distinct yet interrelated phases: radiation pneumonitis (RP) in the early phase and radiation fibrosis (RF) in the late phase. The mechanisms underlying RILI are complex, involving numerous cellular and molecular interactions that lead to the accumulation, proliferation, and differentiation of fibroblasts, ultimately resulting in excessive deposition of extracellular matrix and pulmonary fibrosis ultimately (13). Radiation-induced brain injury (RIBI) has become a severe complication after radiotherapy in patients with head and neck malignant tumors, characterized by headache, fatigue, lethargy, and attention deficits at the early phase after radiotherapy, as well as irreversible cognitive dysfunction in 50-90% of patients who survive >6 months (14, 15). Previous research has demonstrated that ionizing radiation affects the production of proinflammatory factors, neuroin-inflammatory signaling cascades, gliosis, and damage to endothelial cells, ultimately leading to impairments in hippocampal function (16, 17).

In addition, radiation-induced liver disease (RILD) is also a concerning issue with a high mortality rate. RILD can be categorized into two types: classical RILD (patients without underlying hepatic disease) and non-classical RILD (patients with underlying hepatic disease). Patients with classical RILD typically present with fatigue, rapid weight gain, increased abdominal girth, hepatomegaly, anicteric ascites, occasional right upper quadrant pain, and isolated elevation of alkaline phosphatase out of proportion to other liver enzymes. Conversely, patients with non-classical RILD present with markedly elevated transaminases and jaundice (18, 19). The morphologic appearance of patients with RILD is that of endothelial swelling, veno-occlusive disease, sinusoidal congestion, atrophy of liver parenchymal zones and collagen deposition (20). With respect to the gastrointestinal tract, nearly all patients who undergo abdominal and pelvic radiotherapy suffer from gastrointestinal symptoms. Clinically, nausea, vomiting, diarrhoea tenesmus, abdominal pain and incontinence may occur during initial fortnight after radiotherapy. Delayed symptoms of radiation gastrointestinal injury occur few months to years following radiotherapy with complications of diarrhoea, recurrent abdominal pains, malabsorption, perforation, fistulation and bleeding (21, 22). In summary, ionizing radiation induces tissue damage across multiple organs, with severity and manifestation depending on both the radiation dose and the inherent radiosensitivity of the affected tissues.

## 3 Immune cells involved in radiation-induced injury

### 3.1 Macrophages

Macrophages, as innate immune cells, play key regulatory roles in the repair of tissue integrity and maintenance of tissue homeostasis. Characterized by their heterogeneous phenotypes and functions, macrophages can be activated by the surrounding microenvironment, such as cytokines, growth factors, microbial products, nucleotides, and various other modulators. Macrophages typically polarize into two distinct phenotypes: classically activated macrophages (M1) and alternatively activated macrophages (M2). In response to inflammatory cytokines such as TNF-α and IFN-γ, M1 macrophages mediate the acute inflammatory responses and participate in Th1 immune reactions by releasing pro-inflammatory cytokines and chemokines including IL-1, IL-6, IL-12, IL-15, IL-23, CCL2, CXCL10, and TNF-α. Conversely, M2 macrophages are responsible for resolving wound healing processes, downregulating inflammation and promoting fibrosis through the secretion of TGF-β, IL-10, CCL17, CCL18, and Arg-1 (23, 24).

The microenvironmental factors that are altered in tissues after radiation exposure have a significant impact on the dynamics of macrophages, thereby influencing their activated phenotypes and function. During the acute stage of IR response, macrophages tend to polarize towards the pro-inflammatory M1 macrophages, triggering inflammatory responses that facilitate the development of radiation-induced injury. In the later stage of the ionizing radiation injury, the dominant phenotype of the macrophage undergoes a shift from the pro-inflammatory M1 type to the pro-fibrotic M2 type. Once activated, M2 macrophages release immunosuppressive factors that reduce inflammation and promote fibrosis (25, 26).

Cellular senescence is a state that occurs when previously proliferation-competent cells undergo irreversible cell cycle arrest, accompanied by marked morphological changes and characteristic gene expression patterns, in response to multiple intracellular stresses (27, 28). Previous studies showed that ionizing radiation (IR) could induce the senescence of bone marrow-derived monocytes/macrophages (BMMs) with the increasing expression of senescence-associated β-galactosidase (SA-β-Gal) and senescence-specific genes (p16, p21, Bcl-2, and Bcl-xl). Besides, IR could promote the secretion of pro-fibrogenic factors (TGF-β1 and Arg-1), senescence-associated secretory phenotype (SASP) proinflammatory factors (Il-1α, Il-6, and TNF-α), SASP chemokines (Ccl2, Cxcl10, and Ccl17), and SASP matrix metalloproteinases (Mmp2, Mmp9 and Mmp12), which might contribute to the development of pulmonary fibrosis (29). In addition, radiation-induced macrophage senescence is related to the risk of atherosclerosis (30).

Triggering receptor expressed on myeloid cells (TREM), a member of the Ig-like immunoregulatory receptor family, functions as a critical amplifying molecule of inflammation, leading to dysregulation of innate immune system in a number of inflammatory disorders and contributing totissue damage and poor prognosis (31). Previous research demonstrated that radiation upregulates TREM-1 expression in mouse macrophages *in vitro* and *in vivo* by the release of extracellular Cold-inducible RNA-binding protein (eCIRP). Moreover, knockout of TREM-1 remarkably increased 30-day survival after total body irradiation in mice (32). These results suggested that targeting TREM-1 could lead to the development of a potential novel approach for radiation injury. In summary, macrophages play a dynamic role in radiation-induced injury, with their phenotypic shift from pro-inflammatory M1 to pro-fibrotic M2 types driving both acute damage and chronic fibrosis. Additionally, radiation-induced macrophage senescence and upregulation of TREM-1 further exacerbate tissue injury, highlighting the potential therapeutic targets for mitigating radiation effects.

### 3.2 T Helper cells

Upon activation, naïve CD4+ T cells differentiate into distinct subsets of T helper (Th) cells with specialized functions, which are defined based on a unique network of transcriptional regulators and characteristic cytokine profiles. Different T helper cell subsets, with intricate network of inflammatory signaling pathways, play a key role in orchestrating the immune response, with a crucial function in infection, inflammation, and autoimmunity (33). There are numerous pieces of evidence that suggest alterations in the balance among CD4+ Th cells are involved in the onset and progression of radiation-induced injury.

Th1 cells, as one subtype of CD4+ T helper cells, characterized by the production of cytokines such as IFN-γ to suppress fibroblast activity and attenuate collagen deposition (34). Exposed to ionizing radiation, the excessive production of pro−inflammatory factors, such as IFN-γ and IL-2 by Th1 cells, aggravates the clinical side effects of radiotherapy, including radiation-induced lung and intestinal injury, radiation encephalopathy, as well as other complications. On the other hand, cytokines such as IL-4 and IL-13 produced by Th2 cells play significant roles in enhancing fibroblast activation and stimulating collagen synthesis (35). Compared to wild-type mice, Th1-deficient mice exhibit an increased fibrotic response to radiation, characterized by elevated expression of TGF-β1, COL3A1, and potentiated irradiation-induced collagen deposition in the mucosa. In this intestinal irradiation model with Th1-deficient mice (T-bet-/-), the severe lack of transcription factors and cytokines characterizing the Th1 lineage and the exacerbated Th2-like immune response take part in the development of the intestinal collagen deposition (36). Han et al. reported that the Th2-like immune response contributes to the progression of radiation-induced pulmonary fibrosis (RPF), with the Th2 cell-centric transcription factor GATA-3 serving as a crucial factor in this immune response (37). In general, an exaggerated Th1 immune reaction primarily contributes to the development of inflammation, whereas an overactive Th2 immune response predominantly plays a role in the process of fibrosis.

Distinct from Th1 and Th2 cells, the activation of an independent transcriptional program drives the differentiation of CD4+ naïve T cells into Th17 cells, enabling their specific biological functions. Th17 cells have been revealed as critical members of auto-immunity and inflammation (38). The specific function and underlying mechanism of Th17 cells in radiation-induced injury are still unclear. Bessout et al. reported that Th17 cells were generated and accumulated in the irradiated intestines of mice. IL-17 directly stimulates colonic smooth muscle cells to express pro-inflammatory genes *in vitro*, which may contribute to the development of radiation-induced injury. Additionally, IL-17 could contribute to the persistence of impaired tissue regeneration and fibrosis following irradiation. Adipose mesenchymal stromal cells (Ad-MSCs) treatment suppressed the Th17 population for reducing late side effects induced following irradiation (39). Yet, in a contradictory finding, Huang et al. reported that IL-17A plays a significant role in radiation protection. Scleroglucan may enhance the proliferation and regeneration of intestinal stem cells (ISCs) by activating intestinal immune response mediated through the IL-17 signaling pathway, thereby exerting a protective effect against radiation-induced injury (40). Bacterial dysbiosis served as a feature of radiation enteritis. To defend against bacterial offence, the accumulation of Th17 cells in the damaged gut serves as a feedback mechanism to elevate local IL-17 levels, which stimulates residual epithelial cells to increase the production of defensins. Additionally, Th17 cells exhibit anti-infective properties by secreting granulocyte-macrophage colony-stimulating factor (GM-CSF), which promotes the proliferation of recruited neutrophils and monocytes, aiding in the elimination of damaged cells and pathogens. However, this process also exacerbates oxidative stress post-irradiation, compromising cell survival and ultimately fostering an inflammatory environment in the irradiated gut (41). Numerous studies have supported the idea that ionizing radiation-induced cellular senescence promotes tissue fibrosis following radiation (42). It has been reported that CCR6+Th17 cells exhibited a high sensitivity to IR-induced senescence, which in turn promoted the secretion of IL-8 and VEGF-A. This process may contribute to IR-induced damage in normal tissues and potentially facilitate tumor recurrence and metastasis following radiotherapy (43). In addition, several studies proposed that the activation of Th17 cells might be instrumental in the development of pulmonary fibrosis. Paun et al. reported that the acute adaptive immune response correlated with late radiation-induced pulmonary fibrosis in mice. At the 7 day timepoint following radiation, greater numbers of Th17 cells were significant predictors of late stage pulmonary fibrosis (44). According to studies using mouse models, the irradiated IL-17−/− mice lacked Th17 cells, were protected from both fibrosis and pneumonitis, allowing them to exhibited prolonged survival (45).

Regulatory T cells (Tregs), a subset of CD4+ T cells, have long been emerging as major regulators of inducing peripheral tolerance, inhibiting aggravated inflammation and mediating tissue recovery. A growing body of evidence has demonstrated that Tregs play suspected differential roles in the acute pneumonitis and pulmonary fibrosis of RILI (46). Liu et al. found that Tregs inhibited lung inflammation after the silica exposure, while the depletion of this cell population caused a delay of the lung fibrosis by shifting toward a Th1-dominated pro-inflammatory response (47). It is tempting to speculate that the role of Tregs in radiation-induced tissue injury may also be similar and requires further exploration. A murine study demonstrated a transient accumulation of local and systemic cells with immunosuppressive CD4+ FoxP3+ Tregs at 21 days whole thorax irradiation. Furthermore, Tregs isolated at this time point have normal immunosuppressive function which are able to control effector T cells with tissue-destructive activity including Th1 andTh17 cells (48). Therefore, Tregs may play a significant role in protecting normal tissues from excessive inflammatory damage in the early pneumonitis phase.

Intriguingly, current evidence has emphasized the key participation of Tregs in accelerating the progress of radiation-induced pulmonary fibrosis. During the fibrotic phase of pulmonary injury, Wirsdörfer et al. documented that thoracic irradiation triggers the accumulation of CD4+Foxp3+ Tregs in the lungs (48). Another murine study indicated that the long-term (six months) depletion of Tregs by anti-CD25 antibodies lessened the severity of lung fibrosis in mice (49). Of note, this approach may also deplete CD8+CD25+ T-cells, and the reduction of such CD8+ T-cell subsets might likewise contribute to the observed attenuation of lung fibrosis (50).

Several potential mechanisms could explain the contribution of Tregs in exerting a pro-fibrotic role in RPF. First, an increased number of fibrocytes are involved in the radiation-induced lung injury during both the acute and chronic phases of RILI, playing an important role in pulmonary fibrosis. Tregs may affect fibrocytes by promoting their accumulation in irradiated lungs. Supporting this hypothesis, Xiong et al. found that the depletion of Tregs diminished irradiation-induced rise in the number of fibrocytes in mouse lung tissues attenuating lung fibrosis (49). The potential mechanism may be related to the Treg-mediated release of TGF-β, which is known to promote the fibroblast proliferation, differentiation, and collagen synthesis (51, 52). Second, Tregs can promote epithelial-mesenchymal transition (EMT) in accelerating irradiated pulmonary fibrosis. EMT, a cellular process relevant to the transdifferentiation of epithelial cells into motile mesenchymal cells, is known to be crucial for embryonic development and fibrotic tissue repair including pulmonary fibrosis (53). Previous study suggested that radiation induced β-catenin, a key indicator of EMT, aberrant accumulation in the alveolar epithelium. The coculture of irradiated mouse lung epithelial (MLE) 12 cells with Tregs suggested that Tregs promoted the EMT process and the effect of Tregs on EMT was partially hindered in irradiated MLE 12 cells with β-catenin knockdown (54). Additionally, the interaction between Tregs and other immune cells may also accelerate the development of RPF. Evidence suggested that Treg depletion disrupted the balance of Th1/Th2 shifting in favor of Th1 dominance with a cytokine profile of augmented Th1 cytokines (IFN-γ and IL-12) and decreased Th2 cytokines (IL-4 and IL-5) Another potential mechanism is that Tregs accelerate RPF by attenuating Th17 response and Th17 may play a potent anti-fibrotic role in RPF (49). Collectively, these findings suggest that Th cells play an important role in radiation-induced injury. Their dynamic interplay regulates the balance between inflammation and fibrosis, shaping both acute damage and long-term tissue repair.

### 3.3 Neutrophils

Neutrophils, the most abundant circulating cells in the innate immune system, are major pathogen-killing phagocytes. For many years, neutrophils have been shown to be the first leukocytes to arrive at the sites of infection to defend various infectious and inflammatory diseases. However, the excessive infiltration of neutrophils could promote tissue and organ damage by releasing harmful inflammatory mediators, including cytokines, proteases, and reactive oxygen species (55).

Compared with lung and liver tissues, RNA sequencing indicated that the expression of characteristic genes of neutrophils was most significantly increased in intestinal tissue after irradiation. Compared with the non-irradiation group, the results of H&E staining displayed obvious deterioration of intestinal lesions within the irradiation group. Specifically, these lesions contained structural devastation of intestinal crypts, hyperemia and edema of the intestinal mucosa, along with a substantial elevation in neutrophil infiltration. Interestingly, neutrophil depletion by anti-Ly6G antibody attenuated intestinal injury in mice, revealing a detrimental role of neutrophils in radiation enteritis. Although the underlying mechanisms remain unclear, the promotion of irradiation-induced intestinal injury mediated by neutrophils may be related to CXC motifchemokine receptor 2 (CXCR2), which is known to mediate the migration of neutrophils to the site of inflammation and injury by combining with its ligands. Upon exposure to ionizing radiation, CXCR2 and its ligands were significantly upregulated in intestinal tissue. Moreover, the infiltration of neutrophils and intestinal injury were reduced by the CXCR2 antagonist SB225002, suggesting that CXCL-CXCR2 axis is a potential mechanism whereby neutrophils induce radiation enteritis (56).

In the radiation-induced lung injury, a clinical study reported that neutrophil counts were increased in cancer patients undergoing thoracic radiotherapy. Furthermore, another murine study demonstrated that neutrophil infiltration accumulated contribute to the development of radiation-induced lung diseases, including pneumonitis and fibrosis. Fox et al. found that radiation induced alterations in the pulmonary expression levels of CXCL1 and CXCR2, which play a role in the recruitment and activation of neutrophils. Additionally, the neutrophil elastase inhibition decreases radiation-induced alveolitis and prolongs survival in mice (57).

In a multivariate predictive model, D. De Ruysscher et al. found that the occurrence of maximal neutropenia during concurrent chemoradiation in lung cancer patients was notably correlated with the higher incidence of acute radiation-induced dysphagia. Several possible mechanisms may contribute to the elevated incidence of dysphagia in patients with severe neutropenia. It is possible that neutrophil granulocytes are essential for safeguarding and restoring the esophageal mucosa. Patients with low neutrophil counts may be more prone to infections such as fungal infections in their injured esophagus, potentially resulting in severe dysphagia. It is tempting to speculate that neutrophil growth factors could potentially be beneficial for the recovery from radiation-induced dysphagia. Moreover, Severe neutropenia could indicate a significant depletion of bone marrow stem cells, which might be crucial for the restoration of radiation-induced esophageal damage (58). While neutrophils exacerbate intestinal and lung damage through CXCR2-mediated inflammation, their depletion can impair esophageal mucosal recovery, potentially due to impaired mucosal defense and repair. Therefore, therapeutic strategies targeting neutrophils must balance the detrimental inflammatory effects against their protective roles in tissue recovery and infection control.

### 3.4 Dendritic cells

**Figure 2:**
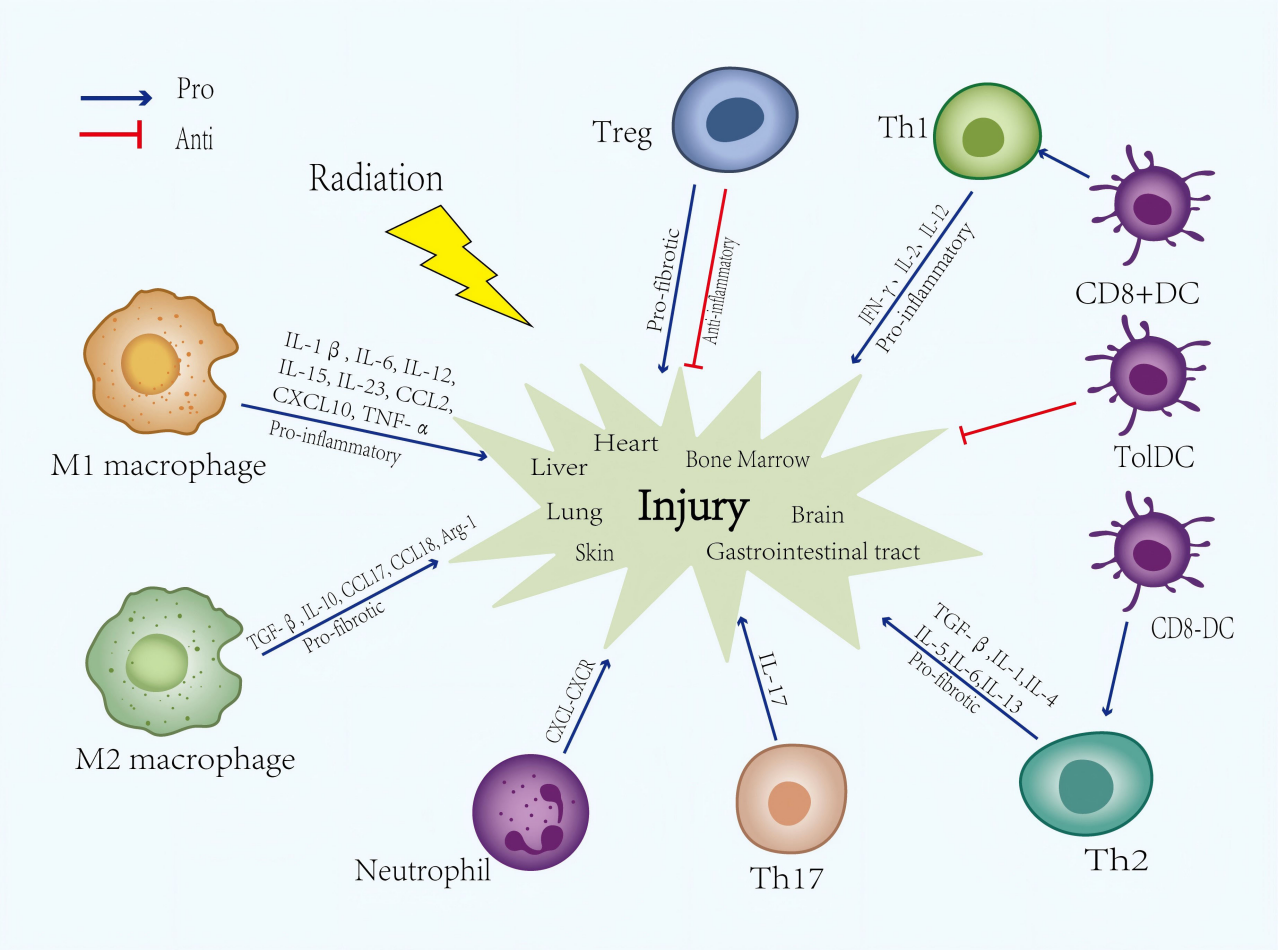
Immune cells and cytokines, chemokines involved in the radiation-induced injury. They play pivotal roles in initiating inflammatory responses and influencing tissue repair and recovery processes following radiation exposure. Through complex signaling pathways, they collectively regulate the response mechanisms to radiation injury. IFN-γ, Interferon-γ; IL, Interleukin; Th, T helper cell; DC, dendritic cells.

DCs are considered as the most efficient antigen-presenting cells (APCs) that link both innate and adaptive immune system function, orchestrating T cell immunity and tolerance (59). Ionizing radiation disrupts the equilibrium between effector immunity and regulatory immunity within lung tissue, thereby eliciting alterations in the behavior of DCs as a consequence of these disruptions. DCs are categorized into several subpopulations with specialized functions, such as plasmacytoid DCs (pDCs) and conventional DCs (cDCs). Moreover, cDCs can be divided into CD8- and CD8+ subsets (60). Distinct subsets of dendritic cells (DCs) elicit specific types of T cell responses. Specifically, CD8+ DCs have been shown to primarily stimulate the development of Th 1 type immune response, whereas CD8- DCs induce the Th2 type immune response (61). A previous study found that radiation might lead to Th1/Th2 imbalance through downregulating the ratio of CD8 +/CD8- DC, which was reversed by Flt3 ligand pretreatment, providing a novel mechanism of radiation-induced immunosuppression (62). DCs that exhibit a stable semi-mature phenotype as well as tolerogenic properties are classified as tolerogenic DCs (tolDCs). Previous studies have shown that tolDCs play a pivotal role in maintaining immune tolerance and preventing lung tissue from damage caused by excessive immune response-induced (63). TolDCs are capable of interacting with Tregs and Bregs, thereby promoting the generation of immunosuppressive molecules (64). Thus tolDC-based therapy can be a promising choice in treating RILI (**Figure 2**). These findings suggest that radiation disrupts the equilibrium of dendritic cell subsets, contributing to Th1/Th2 imbalance and immune dysregulation in lung injury. Additionally, the induction of tolerogenic DCs presents a promising therapeutic strategy to restore immune tolerance and mitigate radiation-induced pulmonary damage.

## 4 Pivotal cytokines involved in radiation-induced injury

### 4.1 Interleukin-1 family

The IL-1 family consists of eleven proteins, which share a common structural framework, an unconventional secretion mechanism and a similar receptor complex. These cytokines are intricately involved in diverse facets of the tissue inflammatory response. IL-1α, IL-1β, IL-18 and IL-33 belonging to the IL-1 family, are potent pro-inflammatory cytokine and appear to contribute to the development of radiation injury (65). Research by Javed Mahmood and colleagues revealed that in the murine lung, IL-1α expression surged at 4 weeks after radiation exposure in RILI. Subsequently, there was a decline in levels at 8 weeks, followed by no further change until 28 weeks. For IL-1β there was a significant increase following irradiation from 8 to28 weeks compared to the control group (66). Moreover, the observations have suggested that there is a trend toward a decline of circulating IL-1α during RT. IL-1α and IL-6 of blood specimens possess predictive significance in clinical populations who subsequently develop radio-pneumonitis (67). Previous studies suggested that keratinocyte is the predominant IL-1-producing cell after irradiation of the skin and radiation triggers an immediate and sustained increase of the expression level of IL-1β mRNA in the skin tissue of C57BL/6 mice. Interestingly, mice that lack either IL-1 or the IL-1 receptor have a near absence of histopathological reactions after radiation in the skin. It is widely known that the production of type III collagen is sustained by a balance of MMP-related degradation and collagen synthesis under normal collagen structure in skin and other tissues. However, upregulated IL-1 pathways over time in the skin towards overcome the efficacy of MMPs in inhibiting abnormal collagen deposition. These data indicated that IL-1 pathway may be a critical component leading to the pathogenesis of radiodermatitis (68). Mezzaroma et al. indicated that IL-1 blockade limited acute cardiac injury and preserved ventricular contractile reserve and systolic function in mice undergoing low-dose XRT (14 Gy). However, IL-1 blockade failed to rescue the 20 Gy-treated mice from severe myocardial and pericardial fibrosis and premature death. These results implicated that IL-1 is involved in some, but not all, of the mechanisms that contribute to the progression of radiation-induced cardiomyopathy. Furthermore, after radiation exposure for 2 weeks, treatment with the IL-1 receptor antagonist anakinra reduced arterial inflammation in the mouse model of vascular irradiation damage. Notably, both IL-1α and IL-1β are functionally antagonized by the IL-1 receptor antagonist (IL-1RA). In these studies, using IL-1RA knockout mice and the IL-1-blocking agent anakinra made it difficult to discern the distinct contributions of IL-1β and IL-1α to the observed phenotypic changes (69).

IL-18, secreted by monocytes, macrophages, and dendritic cells (DCs), plays a pivotal role in radiation-induced damage and dysfunction (70). Ha et al. reported that increased circulating IL-18 can be used as a proportional indicator of both the severity of total-body irradiation-induced injuries and the magnitude of the radiation dose in various animal models, including mice, minipigs, and non-human primates (71). Additionally, urine levels of IL-18, serving as an early prognostic indicator of survival in irradiated nonhuman primates (Macaca mulatta), enhanced the prediction accuracy of mortality risk stemming from radiation exposure (72). Interestingly, IL-18 knockout mice exhibited significantly greater radiation resistance compared with the wild-type control group when exposed to 9.0 Gy of total body irradiation (TBI). This radiation resistance may be linked to the distinct composition of the gut microbiome in IL-18 knockout mice, which potentially is developed as radiation mitigators (73). The IL-18 binding protein (IL-18BP) serves as a natural antagonist to IL-18. A previous study indicated that the treatment of IL-18BP could protect multiple organs from radiation injuries through IL-18/IFN-γ and/or reactive oxygen species (ROS) inflammatory pathway, especially when treated within an extended therapeutic window starting from 24 hours post-exposure to lethal doses of TBI and beyond. Thus, IL-18BP emerges as a promising therapeutic candidate for managing the devastating consequences of radiation exposure (74). Notably, homogeneous total body irradiation fails to accurately represent the radiation exposure encountered in an actual nuclear incident. To further investigate the role of IL-18, a mouse model of partial body irradiation with 5% bone marrow sparing (PBI/BM5) was developed to mimic the inhomogeneity of realistic nuclear exposure. IL-18 knockout mice demonstrated significantly greater resistance to radiation compared with wild-type mice following PBI/BM5. Significant elevation in serum IL-18 levels was observed only in wild-type mice on day 7. The delayed elevation of IL-18 may partly explain why the short treatment schedule of IL-18BP was not effective against PBI/BM5. In addition, compared with wild-type mice, IL-18 knockout mice exhibited significantly reduced levels of serum IFN-γ on day 14 following PBI/BM5. The results showed that IL-18 played a crucial role in the survival of mice in a realistic nuclear exposure model, possibly via the IL-18/IFN-γ signaling pathway (75).

IL-33, a tissue-derived nuclear cytokine, is widely expressed in endothelial cells, epithelial cells and fibroblast-like cells. Upon cellular injury or tissue damage, IL-33 acts as an intracellular alarmin, triggering a response by alerting immune cells that express the ST2 receptor (IL-1RL1). It plays an important role in maintaining cell homeostasis, orchestrating immune responses, and facilitating tissue regeneration (76). Radiation-induced skin injury (RISI) is one of the most common adverse reactions to radiotherapy. In the healing from the injuries caused by high doses of ionizing radiation, re-epithelialization and collagen deposition play pivotal roles in restoring skin integrity. The accumulation of radiation-induced senescent cells in irradiated tissues is accompanied by active cell proliferation, fibroblast activation, and angiogenesis (77). Through analysis of a publicly accessible single-cell sequencing dataset, Chen et al. found that senescent fibroblasts emerged as the primary cell responsible for eliciting pro-repair effects. IL-33 was one of the key cytokines produced by senescent fibroblasts. IL-33 significantly induced macrophage polarization toward the M2 phenotype, thereby facilitating the repair of RISI and altering the local immune microenvironment towards to the pro-repair direction (78). Furthermore, upon exposure to ionizing radiation, the expression of the IL-33 receptor ST2 on the surface of hematopoietic stem cells (HSCs) in mice was rapidly elevated. IL-33 treatment in turn ameliorated radiation injury in HSCs and apparently improved the survival of irradiated mice. IL-33 overrides DNA damage resulting from radiation exposure by stimulating the early cycling of HSCs. This effect is contingent upon the activation of the PI3K-AKT signaling pathway. These findings suggested that IL-33-induced proliferation of HSCs accelerated the early augmentation of HSC reconstitution, thereby facilitating recovery following radiation (79). Additionally, it is reported that IL-33/ST2 axis serves as a crucial regulator of intestinal regeneration following radiation injury. IL-33 expression was markedly increased in the intestines of WT mice after radiation injury, contributing to the protection of the ISC compartment and regeneration subsequent to radiation-induced injury. In response to IL-33, adjacent Paneth cells increased the production of epidermal growth factor (EGF), thereby enhancing ISC recovery and facilitating epithelial regeneration (80). While IL-1 and IL-18 primarily drive pro-inflammatory responses that exacerbate radiation toxicity, IL-33 emerges as an intracellular alarmin that promotes tissue repair via immune modulation and stem cell protection. These observations suggesting that the complexity of targeting these pathways for therapeutic intervention.

### 4.2 Tumor necrosis factor-α

TNF-α, a pro-inflammatory and apoptotic cytokine produced by activated macrophages during the acute phase reaction, is rapidly and continuously expressed post-ionizing radiation. Abnormal production of TNF-α, when triggered by stressful conditions like radiation, activates the TNF receptor 1 (TNFR1) signaling pathway. This activation is associated with pathological changes, including the development of inflammation, apoptosis, and fibrosis, ultimately resulting in latent function damage (81). Zhang et al. reported that lung radiation transiently induced local production of TNF-α in a mouse model of RP. The inhibition of the TNF-α pathway provides radioprotection in the normal lung and not intrathoracic tumor (82). By exposing genetically modified mice to 10 Gy of ionizing radiation, researchers observed that the absence of TNF-α, TNFR1, and TNFR2 each contributed to attenuating the symptoms of RILI. These findings suggested that TNF-α pathway plays significant roles in the development of radiation-induced lung damage and that the genetic ablation can mitigate the severity of RILI (83). Christin et al. observed that radiation caused the release of TNF-α in liver macrophages. Treatment of irradiated hepatocytes with TNF-α or cell culture supernatants of irradiated liver macrophages significantly increased apoptosis. The addition of TNF-α antibodies could eliminate this effect. This finding suggested that the TNF-α played a significant role in enhancing the apoptotic response of hepatocytes exposed to radiation (84). In addition, a previous study showed that TNF-α in blood samples could serve as a viable biomarker to predict adverse effects following interstitial high-dose-rate (HDR) brachytherapy for liver metastases, including focal radiation-induced liver injury (fRILI) (85). TNF-α has also been identified as a factor involved in the development of radiation-induced oral mucositis (86), enteritis (87), and dermatitis (88). Collectively, TNF-α not only as a potential therapeutic target for mitigating radiation damage but also as a promising biomarker for predicting normal tissue complications following radiotherapy.

### 4.3 Transforming growth factor-β

The superfamily of TGF-β has been demonstrated to be a key mediator involved in the processes of tissue repair and regeneration. There are three highly homologous subtypes of TGF-β in mammals, namely TGF-β1, TGF-β2, and TGF-β3 (89).

TGF-β1 is the most prominent isoform which has been demonstrated as the paramount cytokine responsible for the initiation and progression of radiation-induced fibrosis. Ionizing radiation induces direct DNA injury and the production of ROS. Subsequently, ROS activates TGF-β1 which triggers inflammatory signaling cascades to foster myofibroblast accumulation and extracellular matrix (ECM) synthesis. Moreover, TGF-β1 further elevates ROS levels to intensify the profibrotic signals by the feed‐forward loops and the persistent low grade inflammation sustains these processes (90). Numerous studies have revealed a positive correlation between the intensity of radiation-induced damage and the TGF-β1 levels. The radiation-induced upregulation of TGF-β1 expression has been observed in many crucial organs and tissues, including the lung (91), liver (92), kidneys (93), intestine (94), skin (95), bladder (96), heart (97) and vessel (97). Increased expression of intracellular effectors of TGF-β1 signaling has been reported after radiation, specifically the canonical Smad-dependent pathway. TGF-β1 conveys signals by binding to the receptor complexes composed of type I (ALK1 and ALK5) and type II serine/threonine kinase receptors. This ligand-receptor interaction prompts the phosphorylation of the receptors, subsequently igniting an intracellular signaling cascade that involves the phosphorylation of receptor-regulated Smads (R-Smads), namely Smad2 and Smad3. The phosphorylated R-Smads then pair with Smad4, relocating to the nucleus where they regulate the expression of targeted genes (98).

Liu et al. reported that non-small cell lung cancer (NSCLC) patients with thoracic RT have a considerable risk of experiencing radiation pneumonitis. During three-dimensional conformal radiation therapy (3D-CRT), the circulating TGF-β1 levels were significantly higher in patients with RP than in those without RP. Early elevated circulating levels of TGF-β1 may serve as an independent predictive factor for RP. Notably, the high rate of RP is mainly due to score 1 toxicity (99). Wang et al. validated that a model combining mean lung dose (MLD), baseline IL-8 level, and the TGF-β1 ratio at 2 weeks to baseline (TGF-β1 2w/pre) improved prediction of grade ≥2 radiation-induced lung toxicity compared with MLD alone (100). According to the study on mouse models, conditional *Itgav* knockout and the integrin antagonist cilengitide treatment significantly attenuated RIPF in mice by suppressing the activation of TGF-β1 mediated by αv integrin (101). However, Claudia et al. reported that neither the absolute nor the relative levels of plasma TGF-β1 could serve as a reliable indicator for RP in patients irradiated for advanced NSCLC. Notably, plasma levels of TGF-β1 in patients with advanced NSCLC are significantly influenced by cytokine production within tumor tissues. Consequently, the additional release of TGF-β1 into the blood circulation due to radiation-induced lung injury can be obscured by the variable cytokine production of the tumor, making detection challenging (102).

During the initial phase of radiation-induced liver injury, hepatic stellate cells (HSCs) are activated which is the pivotal link of liver fibrosis. Xiao et al. revealed that the expression of TGF-β1 was increased in radiation-induced injured liver tissues. Radiation triggered HSC activation through the activation of the PI3K/Akt signaling pathway, with TGF-β1 playing a crucial role in this process (92). A previous study suggested that gene therapy blocking TGF-β signaling was capable of effectively mitigating the progression of radiation-induced liver fibrosis that has already been established. Anti-TGF-β intervention prevented TGF-β-mediated oxidative stress and inhibited the activation of hepatic stellate cells, thereby mitigating fibrosis. Additionally, it promoted hepatic compensatory functions by alleviating the TGF-β-mediated inhibition of regeneration (103). Additionally, a prospective validation study has shown that the C−509T allele in TGF-β1 was significantly associated with grade 2 or higher radiation-induced breast fibrosis among patients with early-stage breast cancer, further supporting the role of TGF-β1-related genetic variants in predicting late radiation toxicity (104).

Interestingly, TGF-β3 exhibits promising anti-fibrotic characteristics in contrast to the fibrotic function of TGF-β1. Studies in murine model of radiation-induced pulmonary fibrosis have implicated that TGF-β3 mitigated the progression of radiation-induced pulmonary fibrosis and clearly impeded the migration of fibrocytes towards the lungs. In addition, TGF-β3 treatment might shift the Th1/Th2 balance towards Th1 dominance, effectively inhibiting the progression of pulmonary fibrosis (105). In conclusion, TGF-β1 emerges as a master regulator of radiation-induced fibrosis across various tissues, with its circulating levels may help predict complications in normal tissues. While TGF-β3 exhibits promising anti-fibrotic characteristics.

### 4.4 Interleukin-11

IL-11 is a member of IL-6 family that functions as an anti-inflammatory agent in various inflammation-associated diseases (106). Notably, IL-11 stimulates and promotes the maturation of megakaryocytes within hematopoietic tissues to influence thrombocytopoiesis. Additionally, it was confirmed that IL-11 exerted cytoprotective effects on both gastrointestinal (GI) crypts and hematopoietic progenitors (107). Recombinant human IL-11 (rhIL-11) is a drug in clinical use for reducing thrombocytopenia induced by chemotherapy (108). When administered in combination with granulocyte colony-stimulating factor (G-CSF), IL-11enhances recovery of blood granulocytes and selectively rejuvenates myeloid compartments within the bone marrow of acutely irradiated mice (109).

Boerma et al. articulated that local administration of IL-11 ameliorated early intestinal radiation injury in rats. There are several potential mechanisms for IL-11 exerts its protective effects during radiation enteropathy. IL-11 treatment of irradiated intestine reduced numbers of neutrophils and macrophages and production of a variety of inflammatory mediators including IL-6, IL-12, TNF-α, TGF-β, IL-1β, and nitric oxide (110). Moreover, another study indicated that IL-11 mitigated the occurrence of diarrhea and enhanced intestinal epithelial regeneration in a mouse model exposed to 3 Gy neutron irradiation whole body. IL-11 could enhance cell proliferation post neutron irradiation through both MEK and PI3K-dependent signaling pathways, yet it reduces cell death only via the PI3K-dependent signaling pathway (111). Taken together, IL-11 exerts protective effects against radiation injury through multiple mechanisms, suggesting potential targets for the development of novel therapeutic strategies.

### 4.5 Interleukin-6

IL-6 is a pleiotropic pro-inflammatory cytokine that plays crucial roles in regulating immune, inflammatory responses and the acute phase protein response. IL-6 is secreted by several cellular sources, including T-lymphocytes, macrophages, fibroblasts and type II pneumocytes (112). According to previous studies, the dysregulated expression of IL-6 significantly contributes to numerous diseases activity, such as rheumatoid arthritis, Crohn’s disease and juvenile idiopathic arthritis. Indeed, an increase of IL-6 level has also been observed in both humans and animals with radiation pneumonia. Guo et al. showed that the expression and secretion of serum IL-6 increased significantly in mice exposed to thoracic irradiation (113). However, early intervention with IL-6RA after irradiation alone could not protect against radiation-induced lung injury in mice (114). A possible reason for this finding is that long-term continuous treating of IL-6RA may be crucial for mitigating lung toxicity. As Anscher et al. found that administering a small molecule inhibitor of TGF-β for an extended period (6 months) was more efficacious in mitigating radiation-induced lung toxicity compared to a shorter duration of treatment (3 weeks) (115). Research by Dominique Arpin et al. revealed that early variations in circulating IL-6 and IL-10 levels during radiotherapy were significantly associated with the risk of radiation pneumonitis, with increased IL-6 and decreased IL-10 levels during the first two weeks serving as independent predictive factors (116). It was also found that the level of IL-6 is increased in the bronchoalveolar lavage (BAL) fluid in irradiated lung areas, while IL-6 concentrations remained unchanged in the BAL fluid recovered from the nonirradiated areas (117). Pre-radiotherapy cytokine levels are non-specific due to their susceptibility to various influencing factors. A significant challenge in employing cytokines as biomarkers for radiation-induced lung injury (RILI) is accurately identifying their source, as cytokines are produced not only in normal lung tissue following irradiation, but are also excessively expressed in the tumor cells.

In the acute phase post focal irradiation of the intestine, the increase of serum IL-6 observed is obviously great. Surprisingly, IL6-/- mice exhibited exacerbated intestinal injury in the acute phase following IR, indicating a protective role of IL-6 in mitigating early radiation-induced damage. Furthermore, mice treated with an anti-IL6R antibody developed more severe intestinal fibrosis two months after irradiation, suggesting that blocking IL-6 signaling may contribute to the progression of delayed radiation injury (118). This finding supports the previously postulated theory that IL-6 and its downstream transcription factor STAT3 are essential for the survival of intestinal epithelial cells. Their presence and activity are necessary to maintain the integrity and function of these cells after insult (119). Interestingly, IL-6 and IL-1 signaling are crucial drivers of both the IR-induced inflammatory response and the hyperplasia of keratinocytes. Bioinformatics analysis revealed that irradiation-induced IL-6 signaling co-localized with the upregulation of the senescence pathway, predominantly in epidermal hair follicles, basal keratinocytes, and dermal fibroblasts. Genetic ablation of IL-6 or IL-1R in mice, or molecular inhibition of these cytokines, significantly mitigated the effects of irradiation-induced acute dermatitis (IRIAD) (120). The opposing roles of IL-6 in different tissues highlight the complexity of inflammatory signaling in radiation responses, suggesting that tissue-specific approaches are required for therapeutic modulation of IL-6 signaling.

### 4.6 Interferon

IFN, a cytokine exhibits various effects, including regulating immunity, antiviral and antitumor activities. According to the structural characteristics, cognate receptors, cell type responsible for its production, and biological activities, IFNs are mainly divided into three types. Type I IFNs include IFN-α, -β, -∈, -κ, and - ω. Type II IFNs include IFN-γ. Type III IFNs include IFN-λ (121).

Previous research revealed that hepatocytes released massive double-stranded DNA (dsDNA) following irradiation. This dsDNA triggered cGAS-STING activation in non-parenchymal cells (NPCs), eliciting the production and secretion of IFN-I, accompanying with the damage to hepatocytes. Suppressing the IFN-I signaling pathway through genetic and pharmacological ablation protected against the development of RILD. Furthermore, peri-HCC liver tissues showed significantly increased STING and IFN-β expression compared to non-irradiated tissues. A positive correlation was observed between elevated serum IFN-β post irradiation and the development of RILD in patients. These findings strongly underscored the pivotal role of cGAS-STING-mediated type I IFN release in promoting RILD (122). Interestingly, Leibowitz et al. reported that STING-dependent IFN-β production in the niche drived ISC recovery and regeneration following radiation through compensatory proliferation and DNA damage removal. Mechanistically, this response was dose-dependent: after 9.1 Gy TBI, IFN-β contributed for a minor fraction (~30%) to proliferation at day 4, whereas after 15 Gy TBI, it served as the primary driver of acute crypt regeneration, triggering a 1400% proliferative surge. The results demonstrated that IFN-β might be a valuable therapeutic agent for radiation-induced acute intestinal injury (123).

IFN-γ, a pleiotropic type II IFN, is predominantly produced by activated Th1 CD4+ T cells, cytotoxic CD8+ T cells and NK cells. Studies indicated that IFN-γ play crucial roles in amplifying Th1 responses and inhibit Th2 differentiation and function. IFN-γ can stimulate macrophage polarization to the M1 phenotype which can release an elevated level of inflammatory cytokines and promote inflammatory processes (124). In addition, related studies have reported its marked role in the anti-fibrotic progress by blocking TGF-β/Smads signaling pathway. In the RILI rat model, the IFN-γ level in the interstitial brachytherapy group with superior survival rates was significantly higher than that in the SBRT group (125). Notably, despite sharing molecular and biological similarities with IFN-I and IL-22, IFN-III signaling failed to enhance thymus regeneration following radiation exposure or alleviate the progression of graft-versus-host disease (GVHD) after myeloablative TBI and allogeneic hematopoietic stem cell transplantation (allo-HSCT) (126). Collectively, IFN exhibits different roles in radiation-induced injury across different tissues. Further investigation is warranted to elucidate the specific mechanisms underlying these effects.

### 4.7 Interleukin-4 and Interleukin-13

Both IL-4 and IL-13, which are members of the IL-4 cytokine family, share a chain of IL-4 receptors, biological functions and signaling molecules. These two cytokines are the symbolic cytokines of Th2 cells, which promote the differentiation and function of Th2 and inhibit the activities of Th1 cells (127). In macrophages, IL-4 suppresses the secretion of pro-inflammatory cytokines TNF-α and IL-1β, curtails the capabilities to generate reactive oxygen species (ROS) and nitric oxide synthase (NOS) intermediates of macrophages, and leads to their polarization towards the M2 phenotype (128). Moreover, IL-4 and IL-13 have emerged as pivotal factors in the tissue remodeling and fibrosis processes (127).

In murine models, Groves et al. suggested that IL-4 played a significant role in restoring and maintaining macrophage populations after high-dose single lung radiation exposure. However, the results revealed that loss of IL-4 could not delay or deter fibrosis and alternative macrophage activation in irradiated mice (129). The irradiated lung tissue of wild-type C57BL/6NcR mice exhibited an accumulation of alternatively activated macrophages, increased levels of IL-13, and widespread fibrosis. IL-13 knockout mice were found to be resistant to the development of radiation-induced fibrosis (130). Also, evidence clearly suggested there was an upregulation of IL-13 mRNA levels in lamina propria NK cells after irradiation of WT mice. IL-13 has the capability to induce intestinal tissue damage. Furthermore, treatment with IL-13Rα2-Ig enhanced epithelial cell regeneration from radiation–induced small intestinal injury (131).

### 4.8 Interleukin 17

IL-17, primarily generated by Th17 cells, is a pro-inflammatory cytokine involved in the pathogenesis of multiple diseases. IL-17 stimulates a series of immune cells to secrete inflammatory mediators and chemokines, and its expression is regulated by cytokines including TGF-β, IL-6, TNF-α, and IL-1β, which have been implicated in radiation-induced injury (132). Xiong et al. reported that IL-17 expression was increased in mice after thoracic radiation (49). Additionally, treatment with an IL-17A antibody alleviated radiation-induced pneumonitis and subsequent fibrosis in mice, enhancing post-radiation survival (133). Research by Paun et al. reported that mice lacking IL-17 were protected from both radiation-induced pneumonitis and fibrosis, allowing them to survive until the end of the experiment (45). In a murine model that closely resembles human radiation-induced oral mucositis (OM), RNA sequencing showed increased expression of IL-17 and associated immune pathways following head and neck irradiation (HNI). Strikingly, IL-17 receptor (IL-17RA) deficient mice exhibited obviously more severe OM, with excessive inflammation and damage to the mucosal layer. Deficiency in IL-17RA results in amplified inflammatory cytokine/chemokine responses, enhanced immune cell infiltration, and severe mucosal injury. Additionally, loss of IL-17RA impairs epithelial proliferation and elevates apoptosis, compromising mucosal integrity. IL-17RA therefore limits tissue damage and supports mucosal repair following irradiation, with important implications for Th17 pathway-targeted cancer treatments (134) (**Table 1**). More attention should be paid to the dual role of IL-17 in radiation-induced injury.

**Table 1:** Summary of studies analyzing radiation-induced cytokine expressions from animal models and clinical models after radiation.

Cytokines	Models	Types of RT	Sources	Doses	Materials	Ref.
IL-1α	Rats/patients	Thorax	X-ray/CXRT/RT	10 Gy/63 Gy	Lung/plasma	(66, 67)
IL-1β	Rats	Thorax/right hind limb	X-ray/^137^Cs γ-ray	10 Gy/30 Gy	Lung/skin	(66, 68)
IL-1	Mice	Thorax	X-ray	14 Gy/20 Gy	Heart	(69)
IL-18	Mice/gottingen minipigs/rhesus macaques/hesus monkeys	TBI/PBI/BM5	^60^Co/X-ray	5 Gy- 16 Gy	Serum/spleen/thymuse/liver/lung/BM cell/urine/peripheral blood/faecal pellet/whole blood serum/heart	(71–75)
IL-33	Mice	Right leg/TBI	X-ray	6 Gy/9 Gy/10 Gy/60 Gy	Skin/BM cells/small intestine	(73–75)
TNF-α	Mice/patients/hamsters/rhesus macaque	Thorax/liver/buccal pouches/TBI	X-ray/ brachytherapy/ ^60^CO	5 Gy- 35 Gy	Lung/plasma/oral mucosa/jejunum, ileum and colon	(71–75, 86)
TGF-β1	Patients/rats	Thorax/abdomen/whole-liver	Various types of RT	Various doses	plasma/liver/blood	(91, 101)
TGF-β3	Mice	Thorax	^60^CO	20Gy	Lung tissue/BALF	(104)
IL-11	Mice/rats	TBI/small bowel	^137^Cs/X-ray/neutron	Various doses	Blood/intestine	(73–75)
IL-6	Mice/patients	Thorax/intestine	X-ray/3D-CRT	18 Gy- 72 Gy	Lung tissue/plasma/serum/BALF/intestine/blood	(71–75, 117)
IFN-β	Mice/patients	Liver/TBI/ABI	^137^Cs/X-ray	Various doses	Liver/serum/intestine	(121, 122)
IFN-γ	Female SD rats	Thorax	X-ray	30 Gy	Lung tissue/serum	(124)
IFN-III	Mice	TBI	X-ray	C57BL/6 5.5 Gy, 11 Gy; Balb/c 4.5 Gy, 9Gy	Thymus/intestine	(125)
IL-4	Mice	Thorax	^137^Cs/γ-ray	12.5 Gy	Lung tissue	(128)
IL-13	Mice	Thorax/TBI	X-ray	6 Gy/3 Gy, 12 Gy	Lung tissue/serum/BALF/intestine	(129, 130)
IL-17	Mice	Thorax/head and tongue	^60^CO γ-ray/X-ray/^137^Cs	15 Gy/18 Gy/18–19 Gy	Lung tissue/BALF/tongue	(45, 49, 132, 133)

IL-x, interleukin-x; Gy, gray; RT, radiotherapy; 3D-CRT, 3D conformal radiation therapy; CXRT, concurrent chemoradiation therapy; TBI, total body irradiation; ABI, abdominal irradiation; TGF-β1, transforming growth factor β1; TNF-α, tumor necrosis factor-α; IFN, interferon; G-CSF, granulocyte colony stimulating factor; BALF, bronchoalveolar lavage fluid.

## 5 Therapeutic strategies

Radiation countermeasures are designed to safeguard organisms from the detrimental impacts of radiation and minimize tissue damage. Based on the time of administration relative to radiation exposure, radiation countermeasures are fundamentally grouped into three classes: radioprotectors, radiation mitigators, and radiation therapeutics. There are various countermeasures stemming from diverse sources, including man-made synthetic chemicals, natural compounds, plant extracts. In the past decades, antioxidants, haemopoietic growth factor, cytokines, immunomodulatory agents, and biostimulants have been tested for mitigating side effects from radiation exposure. In addition, radiation countermeasure strategies based on cellular and gene therapies have also been gradually developed (4, 5). Several promising radiation countermeasures of agents and possible mechanisms are exemplified in **Table 2**.

**Table 2:** Several promising radiation countermeasures of agents and possible mechanisms.

Natural or synthetic	Agent	Proposed mechanism	Ref.
Natural	Curcumin	Decrease inflammatory and fibrogenic cytokines (such as IL-1, IL-6, TNF-α and TGF-β).	(135)
Synthetic	Monophosphoryl lipid A	Promote the polarization of macrophages towards the M1 phenotype.	(136)
Natural	Huanglian Jiedu plaster	Inhibit HMGB1-mediated crosstalk between macrophages and epithelial cells.	(137)
Natural	Nocardia rubra cell-wall skeleton	Promote the phagocytosis and migration ability of peritoneal macrophages	(138)
Natural	Lactobacillus rhamnosus GG	Release lipoteichoic acid, migrate mesenchymal stem cells and activate macrophage.	(139)
Synthetic	Azithromycin	Induce the regulatory function of macrophages, reduce neutrophils influx, and inhibit the pro-inflammatory cytokine expression.	(140)
Synthetic	Soy isoflavones	Inhibit the activation of macrophages and neutrophils.	(141)
Natural	18β-Glycyrrhetinic acid	Suppress the secretion of TGF-β1 from Treg cells, prevent the induction of EMT and the transformation of MFBs.	(142)
Natural	Jiawei Maxing Shigan Tang	Modulate Tregs, decrease the expression of TGF-β1, and inhibit the TGF-β1/Smad signaling pathway and EMT.	(143)
Natural	Bee venom phospholipase A2	Increase the population of Tregs.	(144)

IL-x, interleukin-x; TNF-α, tumor necrosis factor-α; TGF-β, transforming growth factor β; EMT, Epithelial-mesenchymal transition; HMGB1, high mobility histone B1.

## 6 Conclusions

Radiation-induced injury epitomizes a multifaceted biological process, wherein numerous functionally diverse immune cell populations participate in its pathogenesis. Current research indicates that immune cells exhibit multifaceted functions in radiation-induced injury, potentially due to the dynamic alterations in the microenvironment during disease progression. Furthermore, the majority of immune cell types are heterogeneous, with their functional plasticity being modulated by both systemic and local microenvironmental factors. Consequently, the cells play a crucial role in determining their functions injury. Some advancements have been achieved in understanding the cellular and molecular mechanisms of radiation-induced injury. For example, targeting TREM-1 on macrophages has been shown to reduce systemic inflammation and improve survival after total body irradiation by dampening the eCIRP/TREM-1 axis. Activation of the IL-33/ST2 axis skews macrophages toward the M2 phenotype, supports hematopoietic stem cell regeneration, and aids intestinal epithelial repair. For Tregs intervention must be precisely timed because they protect against acute pneumonitis but drive fibrosis in later stages. However, translating these insights into safe and effective therapeutic strategies remains limited. Hence, continued research on the understanding and treatment of radiation-induced injury is imperative.
